# Early Amniocentesis Versus Transabdominal Chorionic Villus Sampling for Early Invasive Prenatal Diagnosis: A 15-Year Prospective Cohort Study

**DOI:** 10.3390/genes17101194

**Published:** 2026-09-26

**Authors:** Petra Podobnik, Tomislav Meštrović, Mario Podobnik, Igor Lončar, Zlata Srebreniković, Dženis Jelčić, Slava Podobnik-Šarkanji, Vesna Benjak, Vesna Vukelić

**Affiliations:** 1 Department of Obstetrics and Gynaecology, Podobnik Special Hospital, 10000 Zagreb, Croatia; petra@podobnik.hr (P.P.); mario.podobnik@podobnik.hr (M.P.); igor.loncar@podobnik.hr (I.L.); zlata@podobnik.hr (Z.S.); djelcic@podobnik.hr (D.J.); slava@podobnik.hr (S.P.-Š.); vesna.benjak@yahoo.com (V.B.); vesna.vuke@gmail.com (V.V.); 2 DA VINCI Polyclinic, Petrovaradinska Ulica 110, 10000 Zagreb, Croatia; 3 University Centre Varaždin, University North, 42000 Varaždin, Croatia

**Keywords:** transabdominal chorionic villus sampling, early amniocentesis, amniovacucentesis, prenatal genetic diagnosis, first trimester, miscarriage, fetal loss, gynecology

## Abstract

Background: Two sampling routes are available when definitive genetic testing is required in the first trimester: early amniocentesis (EA) and transabdominal chorionic villus sampling (TA-CVS). Direct comparisons of the two at 11 to 14 weeks of gestation are, however, few. Our aim was to place the techniques side by side in terms of the genetic information they yield, the chromosomal abnormalities they uncover and the pregnancy outcomes that follow them. Methods: This single-operator, prospective cohort study evaluated singleton pregnancies between 11 and 14 weeks of gestation across 15 years (2010–2024), comparing TA-CVS with EA. Fluid for EA was withdrawn by amniovacucentesis (3–5 mL of amniotic fluid) through a 22-gauge needle, while villus tissue was taken with a 20-gauge needle, both with the ultrasound image visible throughout. Color Doppler assessment of the uteroplacental and fetal vessels and of the fetal heart rate preceded and followed every procedure. Material obtained by either route underwent cytogenetic testing, and targeted copy-number variant (CNV) analysis was added whenever ultrasonography had shown a major structural abnormality. Results: Of 3238 singleton pregnancies, a cytogenetic result was achieved in 1611 of 1613 (99.9%) EA and in 1622 of 1625 (99.8%) TA-CVS cases. An abnormal chromosome finding was present in 138 cases (4.3%), while pathogenic CNVs emerged in 5 of 35 (14.3%) fetuses with structural abnormalities. Spontaneous abortion affected 4 of 1613 EA pregnancies (0.25%) and 3 of 1625 TA-CVS pregnancies (0.18%; difference 0.06%, 95% CI −0.32 to 0.47%; *p* = 0.73). Talipes occurred in two EA cases and none after TA-CVS, whereas hemangiomas occurred in two TA-CVS cases and none after EA. Premature rupture of membranes, preterm delivery, neonatal respiratory distress and neonatal anomalies did not differ between the groups. Doppler pulsatility indices were essentially unchanged after both procedures; small changes reached statistical significance only in the umbilical (*p* = 0.032) and middle cerebral (*p* < 0.001) arteries, and all were transient and unaccompanied by clinical complications. Conclusions: Fetal-derived material adequate for first-trimester genetic diagnosis was obtained by both routes, with cytogenetic results in more than 99% of pregnancies. Beyond the conventional chromosomal abnormalities, targeted CNV analysis added clinically meaningful diagnoses in fetuses with structural anomalies. Invasive fetal sampling therefore retains a definite place in definitive prenatal genetic diagnosis in the era of NIPT.

## 1. Introduction

Invasive sampling of fetal-derived tissue underpins the prenatal diagnosis of chromosomal and monogenic disease, and the first trimester offers two such samples: villi taken from the placenta by chorionic villus sampling (CVS) and amniotic fluid withdrawn before 15 weeks by early amniocentesis (EA) [1,2,3]. Testing at this stage returns an answer sooner than mid-trimester sampling and leaves decision-making to the couple at a more private stage of pregnancy, while for the majority whose result proves normal, it brings reassurance months earlier [4,5]. Which first-trimester route is the safest, and which yields the most dependable diagnosis, therefore bears directly on how prenatal-care pathways are organized and how couples at raised risk are counseled.

Sampling at 15 to 20 weeks remains the yardstick against which invasive prenatal diagnosis is measured, chiefly because its safety record and its diagnostic accuracy are so well documented [6,7]. Villus sampling buys the earliest possible answer, yet brings difficulties of its own—placental mosaicism confined to the placenta and admixture of maternal cells [8,9,10]—and, when undertaken very early, before 9 to 10 weeks, it has been linked to limb-reduction defects and oromandibular hypogenesis [11,12]. Of the two villus-sampling routes, the transabdominal one is generally credited with fewer procedure-related losses than the transcervical [13,14].

The rationale for early amniocentesis was to bring the diagnostic window of amniocentesis forward into the first trimester [15,16,17]. Randomized comparisons with mid-trimester amniocentesis, of which the Canadian Early and Mid-trimester Amniocentesis Trial (CEMAT) has been the most influential, later attributed to EA a greater frequency of fetal loss, talipes equinovarus, leakage of amniotic fluid and failure of cytogenetic culture [18,19], and professional bodies responded by recommending against amniocentesis before 15 weeks [1]. How often complications arise nevertheless turns on how the fluid is taken and on how much of it is removed. Amniovacucentesis withdraws no more than 3–5 mL of amniotic fluid; the needle is kept under continuous ultrasound view and moved as little as possible, so the risk it carries need not be that of conventional early amniocentesis [20,21].

The comparator in most published work is mid-trimester amniocentesis; studies that set EA directly against first-trimester CVS at 11 to 14 weeks are correspondingly few [10,22]. Meanwhile, non-invasive prenatal testing (NIPT) has taken over as the first-line screen for the common aneuploidies [4,23]. Screening is not diagnosis: NIPT returns neither a complete fetal karyotype nor an explanation for a structural anomaly, and it detects copy-number variants unreliably, so confirmation by invasive sampling continues to be required, above all once ultrasonography has flagged an anomaly [24,25,26]. What makes EA and TA-CVS clinically valuable is, accordingly, twofold—how safely they can be performed, and how well the material they provide serves cytogenetic and genomic work, from karyotyping through the assessment of mosaicism to copy-number variant analysis where this is indicated.

Early Doppler studies demonstrated that uterine and umbilical arterial blood-flow velocity waveforms can be assessed in relation to invasive prenatal procedures and during early pregnancy [27,28]. Since the risk attached to any invasive procedure tracks closely with the experience of the operator and with the case volume of the center [13,14], a series drawn from one high-volume operator offers a comparatively clean setting in which to weigh two sampling techniques against each other, the learning curve having been left behind. Our center has sampled chorionic villi for many years [29,30,31], and on that foundation we report a prospective cohort assembled over 15 years (2010–2024) under a single operator, in which EA by amniovacucentesis (3–5 mL) and TA-CVS at 11–14 weeks of gestation are compared for safety and for diagnostic performance. The comparison covers the adequacy of the sample, the success of cytogenetic analysis, the detection of chromosomal abnormalities and of mosaicism, the yield of targeted CNV testing in fetuses with structural anomalies, and the course and outcome of the pregnancies that followed.

## 2. Materials and Methods

### 2.1. Study Design and Participants

From January 2010 to December 2024, a single operator (M.P.) undertook every EA and TA-CVS in singleton pregnancies referred for first-trimester invasive prenatal diagnosis at 11 + 0 to 13 + 6 weeks of gestation, in a monocentric prospective cohort study. Participation was offered, after full explanation, to women who would be 37 years or older at their expected date of delivery and to those referred for any other recognized clinical indication for prenatal diagnosis [1,2]. Eligibility required a confirmed viable singleton pregnancy at 11 + 0 to 13 + 6 weeks of gestation, together with a willingness to attend four scheduled ultrasound examinations: the first at 10–14 weeks and the remainder at 16–20 weeks, at 28–32 weeks and after 36 weeks of gestation. Any woman reporting bleeding, loss of amniotic fluid or abdominal discomfort was seen again without delay. Those who preferred not to enroll were offered other diagnostic options, among them classical MA or late CVS (placental biopsy). Pregnancies that were non-viable on the initial scan, multiple gestations and untreated cervical infection were excluded. Allocation to EA or TA-CVS was not randomized and was not determined by the operator, by placental location, by clinical indication or by calendar period. After structured counseling in which the technique, timing, expected turnaround of the cytogenetic result and reported risks of both procedures were explained, each woman chose the procedure herself, and her preference was recorded before the appointment was scheduled.

Every participant underwent an anomaly scan at 18 to 22 weeks of gestation, with further surveillance scans at 28–30 weeks and after 35 weeks. The study followed the principles of the Declaration of Helsinki and carried the approval of the Ethics Committee of the Podobnik Special Hospital (protocol code 1318, 21 January 2010). Each participating woman gave her consent both orally and in writing, i.e., participants signed the form.

Women were asked to inform the hospital if a pregnancy ended, whether spontaneously or by induction, and those still in the study were telephoned at 20–24 weeks of gestation so that any complication could be recorded. Fetal-loss documentation was completed whenever a spontaneous abortion took place before 24 weeks. Contact continued past 42 weeks until delivery details were available, and for each of the 3238 participants we recorded gestational age at birth, birth weight, and any congenital anomaly apparent on inspection. Neither cap nor quota was applied: every consecutive eligible case entered the cohort, counts were taken from the database, and a predefined primary-indication hierarchy assigned each woman to a single indication so that no case was counted twice. A control cohort of 1655 women whose pregnancies were normal was assembled from consecutive, contemporaneous referrals that reached us through the same pathways and met the same indications, but in whom invasive testing was declined after counseling, usually on personal or religious grounds; the same primary-indication hierarchy governed the coding of their indications. Controls were drawn so as to resemble the study cohort as closely as possible with respect to the obstetric characteristics available in the database, including maternal age, BMI, primary indication and pre-existing obstetric complications. Because assignment reflected the woman’s own decision rather than randomization, exact matching was not achievable and the control cohort remains a self-selected comparator.

Our objective was to gauge how well EA and TA-CVS performed and to identify what raised the risk of spontaneous miscarriage. Miscarriage was accordingly compared between women sampled by either technique and women in whom no procedure was undertaken. The difference in miscarriage rates between these cohorts was used only as an approximate indication of the excess loss observed in women who underwent invasive testing. Because the control cohort was self-selected and only approximately matched, this difference cannot be interpreted as a formal estimate of procedure-related risk. Delivery hospitals supplied the data on pregnancy outcome and on the newborn, including complications arising during pregnancy or labor and findings in the neonate and the placenta.

Relative safety and performance were judged from the complications arising early after sampling—vaginal bleeding, infection, early spontaneous miscarriage, elective termination, abnormal cytogenetic findings, and any fetal loss occurring before 24 weeks of gestation. Maternal age, parity, indication for prenatal diagnosis and gestational age at sampling were comparable across all groups, and the distribution of indications is shown in Table 1. The prospective database was designed to capture the indication for testing, gestational age at sampling, procedural details, the cytogenetic result and pregnancy outcome.

### 2.2. Sampling Techniques and Cytogenetic/Genomic Analyses

For EA the woman lay supine and a single 22-gauge, 90 mm spinal needle was used. The anterior abdominal wall was disinfected, the bladder was left empty, and the needle was advanced under continuous ultrasound control into the central part of the amniotic sac, where 3–5 mL of fluid was withdrawn by the amniovacucentesis technique [21] (Figure 1) [16]. Karyotyping rested on QF-PCR of genomic DNA within 24–48 h together with amniotic cells cultured for seven days.

TA-CVS likewise began with disinfection of the anterior abdominal wall, after which a 20-gauge, 90 mm spinal needle was guided by continuous ultrasound into the central region of the chorion frondosum, the angle of entry being altered by 20–40 degrees where the placental position required it. Tissue was drawn up under negative pressure by the “pistol” technique into a 20 mL syringe already charged with the designated culture medium, as reported elsewhere, and between 5 and 20 mg of chorion frondosum was obtained. Every sample was karyotyped from both a modified short-term culture of 48 h and a long-term culture of seven days. One operator (M.P.) carried out all early invasive procedures; when two passes failed, a further CVS or EA was offered a week later. Amniotic cells and villus tissue alike reached the laboratory promptly and were inspected under a dissecting microscope according to established protocols. Fetal heart rate (FHR) was recorded by M-mode ultrasonography before and after sampling, and anti-D immunoglobulin 50 µg was given to every Rh-negative woman afterwards.

In the first 600 consecutive patients of the series (300 EA and 300 TA-CVS), maternal serum alpha-fetoprotein (AFP) was scheduled 10 min before and 10 min after the procedure to assess feto-maternal hemorrhage; this subset was defined solely by calendar order and not by any clinical characteristic. Paired pre- and post-procedural samples were, in the event, obtained in only 24 of these 600 women, and the AFP analysis is restricted to those paired observations (Table 4). Velocity waveforms in the uteroplacental and fetal vessels were appraised by transvaginal color Doppler. Readings from the uterine artery, the umbilical artery, the spiral arteries, the middle cerebral artery and the ductus venosus were taken 10 min before and 10 min after the procedure on a Voluson E8–E10 (GE Healthcare Austria GmbH & Co OG, Zipf, Austria) system fitted with a 5–9 MHz transvaginal probe, the pulsatility indices (PIs) being computed by the machine.

Each patient was scheduled to be seen in person at follow-up. Details of successful deliveries and of neonatal findings came directly from the hospitals concerned or from the patients themselves, and every record was held electronically. Women managed in 2010 were asked for pediatric reports on intellectual development five years after delivery.

### 2.3. Statistical Analysis

Statistical work addressed the differences between the two techniques in fetal outcome, in the adequacy of the sample and in vascular response. Fetal-loss rates in the EA and TA-CVS groups were compared by a chi-square test of independence, with pairwise Fisher’s exact tests and Bonferroni correction for multiple testing applied wherever an association proved significant. Confidence intervals (95% CI) for differences in proportions were derived by the Newcombe method. Doppler pulsatility indices were compared before and after the procedure separately within the EA and the TA-CVS groups. Paired-sample *t*-tests served to compare the pulsatility index (PI) recorded before the procedure with that recorded afterwards. Significance was set at a two-tailed *p* below 0.05, and all computation was carried out in R version 4.3.3 (R Foundation for Statistical Computing, Vienna, Austria) using base packages.

## 3. Results

The cohort comprised 1613 (49.8%) women who chose EA and 1625 (50.2%) who chose TA-CVS in singleton pregnancies, between 11 + 0 and 13 + 6 weeks of gestation, all sampled by one operator (M.P.). Median gestational age at sampling was 12 + 6 weeks for EA and 12 + 4 weeks for CVS. All procedures in both groups fell within the 11 + 0 to 13 + 6 week window. A reportable cytogenetic diagnosis followed 3233 of the 3238 pregnancies (99.8%), which testifies to the diagnostic adequacy of both routes. Taken separately, QF-PCR of genomic DNA (24–48 h) combined with cell culture (seven days) succeeded in 1611 of 1613 (99.9%) EA cases, while the overnight and long-term culture methods succeeded in 1622 of 1625 (99.8%) TA-CVS cases.

Advanced maternal age (≥37 years) headed the list of indications for both techniques, accounting for 43.0% of CVS and 42.0% of EA cases; the complete distribution appears in Table 1. Early complications, cytogenetic findings, and total fetal loss across the 1613 EA and 1625 CVS procedures are set out by method in Table 2.

Across the whole series fetal loss stood at 4.3% (138 of 3238). Chorionic villus sampling was performed between 11 + 0 and 11 + 6 weeks of gestation in 20% of cases, between 12 + 0 and 12 + 6 weeks in a further 22%, and between 13 + 0 and 13 + 6 weeks in the remaining 58%; no procedure was carried out before 11 + 0 or after 13 + 6 weeks. One insertion sufficed in 96.6% of procedures, the remaining 3.4% calling for a second pass before adequate tissue was secured. Success at first insertion bore no clear relation to gestational age, and every sample collected proved sufficient for diagnostic analysis. Bleeding at some point between sampling and delivery was documented in 32 patients (1.97%), and a placental hematoma of 0.5–1 mL was seen at the biopsy site in 8 cases (0.49%). Bleeding followed TA-CVS (2.0%) twice as often as EA (1.0%), and a peritoneal reaction was encountered after TA-CVS alone. The absolute difference in bleeding between EA and TA-CVS was −0.98% (95% CI −1.86 to −0.14%), and that in abnormal cytogenetic findings was −1.08% (95% CI −2.49 to +0.32%). Neither technique was followed by infection.

A cytogenetic result was available for 3233 of the 3238 cases (99.8%), with tissue proving unsuitable in five (0.2%). Abnormal findings numbered 138 (4.3%). Mosaicism appeared in four TA-CVS cases (0.25%) and in none after EA. In parallel, targeted copy-number-variant (CNV) analysis was undertaken in 35 fetuses in which ultrasonography had disclosed major structural abnormalities, in line with published recommendations [25,26]. Pathogenic CNVs were identified in 5 of them (14.3%): 22q11.2 microdeletion syndrome; 22q11.2 duplication, in two cases accompanied by hydrops fetalis; a 10q26–12q26.3–12q21q22 deletion in a woman of advanced maternal age, 45 years; a 5q21.1–q34 deletion alongside a cardiac anomaly; and 46,XX,del(X) (p11.3p22.3) with hygroma colli, hydrothorax, omphalocele, and hydrops fetalis. Every one of the five pregnancies carrying a pathogenic CNV was electively terminated; none was lost spontaneously. This 14.3% yield refers specifically to the 35 fetuses selected for CNV analysis because of major structural abnormalities on ultrasonography, and it should not be read as a rate for the cohort as a whole. Expressed over all 3238 pregnancies, pathogenic CNVs were identified in 5 (0.15%).

Post-procedure fetal loss, that is spontaneous abortion together with termination of pregnancy, totalled 138 cases (4.3%): 61 (3.8%) following EA and 77 (4.7%) following TA-CVS. Because 131 of these 138 losses (94.9%) were elective terminations performed after an abnormal genetic diagnosis, total fetal loss does not measure procedural safety; the corresponding spontaneous-abortion rates were 4 of 1613 (0.25%) after EA and 3 of 1625 (0.18%) after TA-CVS (difference 0.06%, 95% CI −0.32 to +0.47%; *p* = 0.73). No perinatal death occurred. Spontaneous abortion after CVS became steadily less frequent as gestational age at sampling advanced: 0.6% at 11 + 0 to 11 + 6 weeks, 0.4% at 12 + 0 to 12 + 6 weeks, and 0.16% at 13 + 0 to 13 + 6 weeks. The rate was 1.4% (3 cases) in women younger than 30 years and rose to 4.7% (11 cases) at 40 years and above. Among older women, total fetal loss was more frequent when sampling was performed in the earliest part of the window (11 + 0 to 11 + 6 weeks); when CVS was carried out at 13 + 0 weeks or later, the overall fetal-loss rate fell from 4.5% to 0.15%.

Four randomized studies are set beside the present series in Table 3 with respect to patient numbers, gestational age, chromosomal abnormalities detected, total pregnancy losses, spontaneous losses, congenital abnormalities, repeat testing, and culture failure after EA and TA-CVS.

In the randomized cohort of Nicolaides et al. (1996) [10], spontaneous losses were significantly more frequent after EA than after CVS, the excess amounting to 4.0% (95% CI 0.6–7.7%). Our own series showed no significant difference in total pregnancy losses between the two techniques, and neither did the comparisons of EA with MA reported by Johnson et al. (1996) [32] and Eiben et al. (1994) [16]. Methodology may account for much of the divergence, since the procedure itself, the eligibility criteria, and the gauge of the needle all differ between series. Here, as in the work of Eiben et al. (1994) [16], EA was performed with a 22-gauge needle and no more than 3–5 mL of amniotic fluid was withdrawn. A further distinction lies in the number of operators: several contributed to the studies of Nicolaides et al. [10] and Johnson et al. [32] and three to that of Eiben et al. [16], whereas every procedure reported here was carried out by one (M.P.).

Of the 683 women randomized and followed to completion by Johnson et al. [32], 27 of 344 (7.8%) in the EA arm and 25 of 339 (7.4%) in the MA arm lost the pregnancy, whether spontaneously or by induction (difference 0.5%; 95% CI −3.6 to +4.5%). Spontaneous loss after the procedure came to 2.4% (8/330) with EA against 3.3% (10/299) with MA, a difference that was not significant, and success at the first attempt was 97.6% and 99.7%, respectively. In the 4668 women randomized by Eiben et al. [16], the corresponding losses were 135 of 2866 (4.8%) after EA and 99 of 1802 (5.5%) after MA (difference −0.78%; 95% CI −2.14 to +0.49%), with post-procedure spontaneous loss of 1.0% (29/2866) and 0.5% (9/1802). Perinatal outcome was comparable between the arms of both trials, which is consistent with EA being as safe and as accurate as MA.

Maternal serum AFP and FHR were scheduled before and after the procedure in the first 600 consecutive women of the series. Complete paired pre- and post-procedural measurements were obtained in only 24 of these 600 pregnancies (4.0%), of which three were EA (3 of 300, 1.0%) and 21 TA-CVS (21 of 300, 7.0%); in the remaining 576 women only a single measurement was available, and these were treated as missing data. Table 4 presents the 24 paired cases. A paired AFP value was available in 23 of the 24 (in one pregnancy with a 45,X0 karyotype the AFP samples were unusable) and a paired FHR value in all 24. Mean maternal serum AFP rose from 62.3 ± 23.2 to 99.3 ± 44.9 ng/mL (paired *t*-test, *p* < 0.001), whereas mean FHR was essentially unchanged (140.9 ± 24.5 vs. 140.1 ± 29.1 bpm; *p* = 0.767). No correlation was found between the AFP increase and the volume of chorionic villi aspirated. A chromosomal abnormality was present in 5 of the 24 fetuses (two trisomy 21, one trisomy 13 and two 45,X0), and these five accounted for the most marked changes, with bradycardia in two and tachycardia in three, accompanied by pathological flow in the ductus venosus and middle cerebral artery. Because of the small number of paired observations and the marked imbalance between the two procedures, these findings are descriptive and no comparison between EA and TA-CVS was attempted.

The uteroplacental and fetal vessels were interrogated by transvaginal color Doppler in the 400 consecutive pregnancies of the series (200 EA and 200 TA-CVS) before and after the procedure; the main uterine artery, the spiral arteries adjacent to the sampling site, the intraplacental arteries, the umbilical artery, the ductus venosus and the middle cerebral artery were each examined. Pulsatility indices (PIs) obtained before the procedure were compared with those obtained 10 min afterwards by paired-sample *t*-tests (Table 5). No statistically significant change in PI was observed in the uterine artery (EA 2.10 ± 0.55 vs. 2.11 ± 0.55; TA-CVS 2.11 ± 0.68 vs. 2.15 ± 0.60; *p* = 0.171), the spiral arteries (EA 0.60 ± 0.41 vs. 0.62 ± 0.44; TA-CVS 0.65 ± 0.56 vs. 0.68 ± 0.69; *p* = 0.521) or the intraplacental arteries (EA 0.48 ± 0.15 vs. 0.51 ± 0.22; TA-CVS 0.58 ± 0.26 vs. 0.60 ± 0.17; *p* = 0.638). Small changes reached statistical significance in the umbilical artery (*p* = 0.032) and in the middle cerebral artery (*p* < 0.001); in the latter the change was confined to the TA-CVS group (1.88 ± 0.55 before vs. 1.75 ± 0.60 after), whereas after EA the middle cerebral artery PI was essentially unchanged (1.88 ± 0.55 vs. 1.85 ± 0.58). All pre- and post-procedural values by group are given in Table 5. Most arterial PI values thus held steady, while the umbilical and the middle cerebral arteries registered slight but measurable hemodynamic shifts in the period immediately after sampling. These differences were small in relation to the measurement variability of the technique, were no longer apparent at the subsequent examination, and are thus regarded as transient and of no clinical significance. More specifically, in absolute terms the changes amounted to 0.04 and 0.13 pulsatility index units respectively, which lie within the range of physiological and measurement variability described for these vessels in the first trimester, and neither change was accompanied by an alteration in fetal heart rate, in amniotic fluid volume or in subsequent pregnancy outcome. In every instance the change passed off without any subsequent complication such as spontaneous abortion or preterm rupture of the membranes.

These outcomes were compared with the control cohort of 1655 pregnancies with similar indications and comparable obstetric characteristics, but without any invasive procedure. In this group, the miscarriage rate before 24 weeks was 0.40%, compared with 0.18% (3 of 1625) after TA-CVS and 0.25% (4 of 1613) after EA. Because the control cohort was self-selected rather than randomized, this difference cannot be attributed to the procedures themselves. Follow-up was complete for every case. Fetal anomalies comprised multilocular hygroma colli in 11 cases, cardiac anomalies in 10, genitourinary abnormalities in 9, central nervous system anomalies in 6, universal hydrops in 6, abdominal wall defects in 5 and facial clefting in 1. No serious abnormality of the limbs was seen. Pediatric reports on intellectual development five years after delivery were requested from participants managed in 2010, and no case of intellectual disability was recorded among the records that were returned.

## 4. Discussion

With 3238 singleton pregnancies, this monocentric prospective study ranks among the most comprehensive assessments so far of EA and TA-CVS in the first trimester, the two being compared directly at 11–14 gestational weeks. To the general preference for the abdominal route, our data add the perspective of a single high-volume operator, which takes the learning curve out of the comparison, together with route-specific control cohorts recruited from the same referral stream and the same gestational windows; that design allows excess risk over background to be gauged and keeps procedure-related miscarriage distinct from overall loss, elective terminations after an abnormal result included. Targeted CNV yield is reported alongside pediatric neurology outcomes at five years. Such a combination of elements is seldom found together in the medical literature, and in the Republic of Croatia it is presented here for the first time.

Both techniques proved safe in our hands, with spontaneous abortion, infection and early complications all uncommon in a single center where one very experienced operator did the sampling. Adequacy of the sample was high whichever route was used, and specimen weights kept within the optimal range. Villus yield is nonetheless open to procedural and maternal influences: Alamri et al., in a recent single-center cohort of 240 procedures, obtained larger samples transcervically than transabdominally (37.1 vs. 32.4 mg) and found a raised maternal body mass index to be an independent predictor of a smaller yield [33]. In the present series, one insertion secured sufficient tissue in 96.6% of cases and not a single sample proved inadequate for diagnosis, which suggests that the transabdominal route, in experienced hands, delivers diagnostic-quality material whatever the characteristics of the patient.

Miscarriage became less frequent as gestational age advanced and was least frequent in the youngest women. Doppler interrogation disclosed no major hemodynamic disturbance beyond slight, transient falls in umbilical and middle cerebral artery PI. Chromosomal abnormalities were present in 4.3% of the cohort and pathogenic CNVs in 14.3% of fetuses with structural anomalies. Neither serious limb defect nor intellectual disability came to light at follow-up. Our findings are compatible with the use of TA-CVS as a first-line approach, particularly where procedural volume and expertise are high, and they underline how much turns on appropriate gestational timing and on CNV testing whenever sonographic anomalies are present. This rests on the consistency of tissue yield, on the earlier availability of a result, and on operator familiarity, and not on a demonstrated safety advantage: the difference in spontaneous abortion between the two techniques was 0.06%, which is compatible with no difference at all, and neither technique can be shown to be safer than the other on these data.

For women at heightened genetic risk, EA and CVS are both worthwhile options, and both are ordinarily undertaken between the 11th and the 14th week of gestation. In experienced hands, complications after either are infrequent. A number of comparative studies have examined the safety of CVS against that of EA in pregnancies shown to be viable at the first-trimester scan. The risk of losing the pregnancy has been put at 1–2% higher after CVS than after MA [7,13], though Crane et al. [34] found the miscarriage rates of the two procedures to be statistically indistinguishable. CVS is performed by two main routes, transabdominal (TA-CVS) and transcervical (TC-CVS). One large retrospective cohort of 4862 CVS procedures included 2833 performed transcervically, with forceps in 1787 and a cannula in 1046; loss of pregnancy occurred in 1.4% after TC-CVS overall and in 1.0% after TA-CVS. The disparity within the transcervical group was striking: 0.27% with forceps against 3.12% with a cannula, which points to a possible safety advantage for forceps.

It is instructive to read our results against the randomized evidence that preceded them. The CEMAT trial [18] set EA, performed under continuous ultrasound guidance, against mid-trimester amniocentesis. Of the 3691 patients sampled within the allocated window, 62 lost the pregnancy spontaneously between the procedure and 20 weeks of gestation (EA, 53/1916; MA, 9/1775). EA carried significantly more total fetal loss than MA (7.6% vs. 5.9%; difference 1.7%, one-sided upper CI 2.98%; *p* = 0.012), more talipes equinovarus (1.3% vs. 0.1%; *p* = 0.0001) [19], and more amniotic fluid leakage after the procedure (3.5% vs. 1.7%; *p* = 0.0007); assignment to EA predicted fetal loss, procedural failure, repeated needle insertion, amniotic fluid leakage and culture failure beyond what was seen with MA. Maternal factors reported to raise procedure-related loss include hypertension (fetal loss 11.1% against 2.6% in normotensive women), a raised body mass index and higher gravidity [14].

Pooled analyses help to put these procedure-related risks in perspective. Akolekar et al. [13], reviewing 63,723 amniocentesis procedures, counted 580 miscarriages (weighted loss 0.91%; 95% CI 0.73–1.09%) against a background rate of 0.58% (95% CI 0.47–0.70%), which leaves a weighted procedure-related risk of 0.30% (95% CI 0.11–0.49%). The corresponding figures for CVS were 163 miscarriages after 13,011 procedures (1.39%; 95% CI 0.76–2.02%) against a background of 1.23% (95% CI 0.86–1.59%). Restricting the comparison to pregnancies of comparable baseline risk brought the procedure-related risk down to 0.12% (95% CI −0.05 to 0.30%) for amniocentesis and to −0.11% (95% CI −0.29 to 0.04%) for CVS. Little excess risk, then, is attributable to the procedure itself once it is performed by experienced hands.

Our own 15-year prospective experience yielded lower complication rates than this. The spontaneous abortion rate following TA-CVS was 0.18% (3 of 1625), as against 1.0% in the report cited above. Variation in methodology, in operator experience and in how consistently a standardized technique is applied may all contribute. Sundberg and colleagues [22] set filtered early amniocentesis against CVS and recorded talipes equinovarus in 1.7% of the filtered-EA group and in none of the CVS group, the occurrence of talipes being related to gestational age at sampling. Follow-up over the longer term matters too: Blue and colleagues [35] examined the genetic contribution to pregnancy loss, while Farrell and colleagues [19] found development, behavior, growth and health in childhood to be unaffected by mid-trimester amniocentesis. Taken together, EA between 11 + 0 and 12 + 6 weeks has been linked to more fetal loss, more talipes equinovarus, more amniotic fluid leakage and more cytogenetic-culture failure.

Operator experience also bears on procedure-related pregnancy loss, and roughly 1000 invasive prenatal procedures are carried out at our center each year. Miscarriage was compared with that in a control group in which no invasive procedure was undertaken; in this elective group the miscarriage rate before 24 weeks was 0.40%, compared with 0.18% after TA-CVS. Brambati et al. [36] reported fetal loss before 28 weeks at 2.58% in pregnancies intended to continue, the figure being higher in the older maternal age groups. Miscarriage in our cohort was less frequent in the TA-CVS group. Throughout the past five years TA-CVS has been performed at 13 weeks of gestation, always by the same operator (M.P.). Where specialized centers and experienced operators are involved, no significant excess of miscarriage is apparent, which suggests that the true risk is smaller than is commonly quoted.

Any reading of the pregnancy loss figures has to allow for case mix and for the way cases were ascertained, since these, rather than any harm done by CVS, probably explain why miscarriage was more frequent in some control cohorts than after TA-CVS. Where villi were sampled, serious chromosomal problems came to light early and many such pregnancies were electively terminated, so that a later miscarriage either never happened or was never counted. Comparable high-risk pregnancies among the controls went untested, and a proportion of them ended later in miscarriage, which pushed the control rate upwards.

Aneuploidy and mosaicism restricted to placental tissue, whether to the trophoblast or to the mesenchymal core, are long-recognized phenomena. Short-term chorionic villus cultures in the first trimester yield mosaic results in something like 2–3% of cases, many of which are not borne out by later testing—an awkward finding to interpret in a continuing pregnancy. Long-term villus culture together with fetal karyotyping is therefore advised as a means of resolving such results. Four cases of mosaicism arose in our cohort. Liu et al. [37], investigating the causes of spontaneous abortion, reported a higher proportion, 1.93% of their study population. Our observations are likewise in keeping with a 30-year Croatian cystic-hygroma series [38], in which early sonographic markers were strong predictors of chromosomal pathology and warranted thorough genetic evaluation, a position that recent publications also support [39,40].

A rise in maternal serum AFP after CVS was seen in 8.3% of cases, without any correlation with placental hematoma or with the Doppler findings. Changes in maternal AFP across the procedure pointed to transient feto-maternal transfusion in some pregnancies, and this was related to the weight of chorionic tissue removed. The villous sample was in every case ample for cytogenetic analysis—the consulting laboratory asked for at least 5 mg of tissue for reliable chromosome studies—and feto-maternal hemorrhage of clinical consequence was only rarely detected.

Fetal hemorrhage may in any event disturb embryonic organogenesis through circulatory compromise and hypoxia of the tissues. The systematic review of Akolekar et al. [13] drew on seven studies of CVS and 12 addressing limb abnormalities, which were frequently accompanied by oromandibular hypogenesis and clustered in procedures undertaken while the limbs were at their earliest stage of development; a modest excess of limb abnormalities was apparent where CVS preceded 9 weeks of gestation [11]. No severe limb malformation occurred in our cohort, most probably because sampling was generally done after 12 weeks. As for EA, the amniovacucentesis technique [16,21] removes no more than 3–5 mL of amniotic fluid, under 10% of the total volume, with minimal movement of the needle, and we regard this as the likely explanation for the low miscarriage rate compared with that of techniques that aspirate 20–30 mL.

It should be emphasized that the technique evaluated here is not conventional early amniocentesis. Professional bodies advise against amniocentesis before 15 weeks on the basis of trials in which larger volumes of amniotic fluid, typically 10 to 20 mL, were withdrawn, frequently with repeated needle movement and, in some protocols, with filtration and reinfusion. Amniovacucentesis differs in the volume removed (3 to 5 mL, less than 10% of the first-trimester amniotic volume), in needle gauge (22 G) and in the requirement for a single continuously guided pass with minimal needle excursion. Our findings therefore apply to this specific low-volume technique performed at 11 + 0 to 13 + 6 weeks by a single high-volume operator. They should not be generalized to early amniocentesis as a class, and they do not constitute a challenge to current recommendations concerning conventional early amniocentesis.

### Strengths and Limitations

Among the strengths of this study are a large consecutive series performed by one operator, clearly specified eligibility criteria and the inclusion of route-specific control cohorts. Several limitations nevertheless deserve mention. Since miscarriage risk was compared between intervention and control pregnancies drawn largely from high-risk populations, our estimates may not carry over to large low-risk populations. The influence of operator experience on miscarriage could not be examined at all, one high-volume operator having performed every procedure; external validity is limited accordingly, operator-specific effects cannot be ruled out, and extrapolation to lower-volume or differently organized services calls for caution. Doppler and AFP assessment was confined to subsets of the cohort and to a short interval after the procedure, so delayed or transient effects may have escaped notice, and nothing can be concluded about the transvaginal route. Only fetuses with major structural anomalies underwent microarray/CNV testing, which restricts the generalizability of the yield we report. Because women themselves rather than randomization determined who served as a control, residual self-selection and unmeasured confounding remain possible despite comparable eligibility and referral timing. Furthermore, with only seven spontaneous abortions among 3238 procedures, multivariable adjustment was not feasible and all comparisons are unadjusted. The absence of a statistically significant difference between EA and TA-CVS reflects the very low event rate and cannot be taken as evidence of equivalence or non-inferiority; the study was neither designed nor powered for such a claim, and the appropriate conclusion is that low complication rates were observed with these specific techniques in this specific setting. Finally, only singleton pregnancies managed in a standardized, specialized setting were included, so the findings may not transfer to multiple gestations, to earlier gestational ages or to centers of lower volume. Notably, neither EA nor TA-CVS produced any measurable change in uteroplacental blood flow on Doppler assessment, which makes vascular disruption within the uteroplacental unit an unlikely cause of the fetal losses that followed.

## 5. Conclusions

In this single-operator series, EA and TA-CVS yielded accurate first-trimester genetic diagnoses in high-risk couples and were accompanied by low rates of procedure-related complications. Low and statistically similar rates of spontaneous miscarriage were observed following EA and TA-CVS in this cohort. The low-volume technique may have contributed to the observed low miscarriage rate. Doppler changes following either procedure were transient and bore no relation to adverse pregnancy outcome. Two of these changes reached statistical significance, but their magnitude was small in relation to the variability of the measurement and they had no clinical correlate. The absence of vascular complications in our cohort, coupled with a high diagnostic yield, favors transabdominal early invasive sampling as the approach of choice in early prenatal testing, provided that experienced operators in specialized settings perform it.

These observations describe what was achieved with these techniques in a single specialized, high-volume center. The absence of a statistically significant difference between EA and TA-CVS should not be read as demonstrating equivalence or non-inferiority, and results obtained with low-volume amniovacucentesis should not be extended to conventional early amniocentesis. We would add that the preference expressed here concerns the transabdominal route in general. No statistically significant difference in spontaneous loss was demonstrated between EA and TA-CVS, and neither of the two techniques can be declared superior to the other on the basis of these data. Still, our findings reinforce the continuing place of invasive fetal sampling within contemporary prenatal-genomic pathways, above all where an abnormal NIPT result or a sonographic finding demands a definitive chromosomal or copy-number diagnosis. Confirmation will nonetheless require prospective, standardized multicenter studies that keep procedure-related miscarriage separate from loss due to selective termination. Seen from a public-health standpoint, our results argue for earlier and safer access to dependable prenatal diagnosis, which should improve reproductive counseling, reduce avoidable procedure-related harm and place prenatal screening pathways on firmer evidential ground.

## Figures and Tables

**Figure 1:**
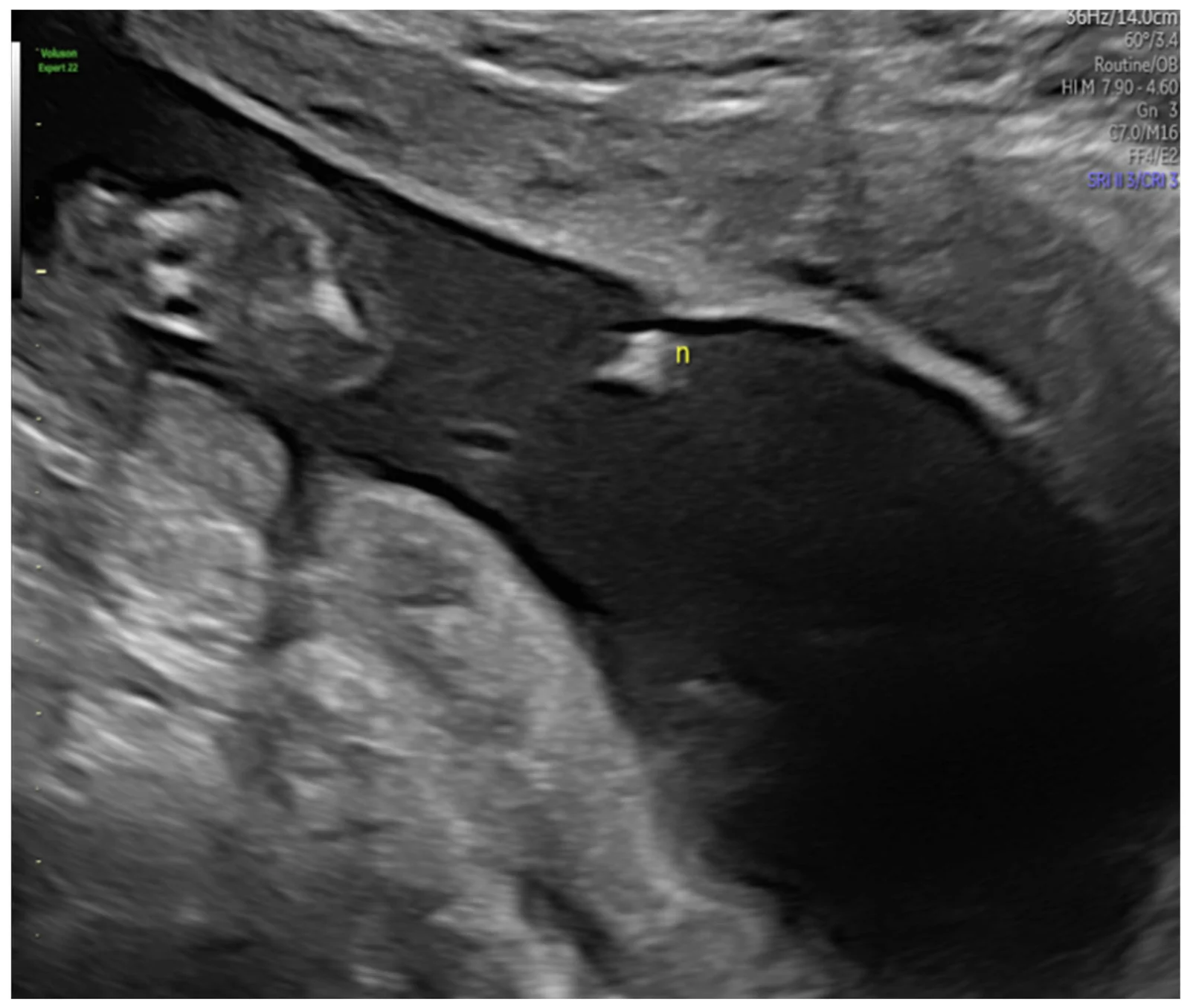
Needle tip (“n”) visible within the amniotic cavity during ultrasound-guided aspiration of amniotic fluid in the first trimester. The image was obtained using conventional grayscale (B-mode) ultrasonography. Dashed lines represent the ultrasound system’s on-screen guidance/measurement markers. Original image by the authors. Original image by the authors.

**Table 1:** Indications recorded for the 3238 first-trimester procedures by technique (EA, early amniocentesis; TA-CVS, transabdominal chorionic villus sampling; NIPT, non-invasive prenatal testing; percentages are calculated column-wise).

Indication	EA n (%)	TA-CVS n (%)	Total n (%)
Advanced maternal age (≥37 years)	678 (42.0)	699 (43.0)	1377 (42.5)
Abnormal ultrasound or NIPT findings	505 (31.3)	521 (32.1)	1026 (31.7)
Previous child with chromosomal anomaly	97 (6.0)	99 (6.1)	196 (6.1)
Risk of metabolic disease	40 (2.5)	39 (2.4)	79 (2.4)
Risk of thalassaemia	25 (1.5)	27 (1.7)	52 (1.6)
Drug or radiation exposure	88 (5.5)	89 (5.5)	177 (5.5)
Family history of intellectual disability	89 (5.5)	91 (5.6)	180 (5.6)
Other or unspecified (e.g., maternal anxiety)	91 (5.6)	60 (3.7)	151 (4.7)
Total	1613 (100)	1625 (100)	3238 (100)

**Table 2:** Early complications, cytogenetic findings and total fetal loss by procedure (total fetal loss = spontaneous abortion + selective termination). Between EA and TA-CVS, the difference in total fetal loss was −0.96% (95% CI −2.37 to +0.44%; Fisher’s exact *p* = 0.19), and the difference in spontaneous abortion was 0.06% (95% CI −0.32 to +0.47%; *p* = 0.73).

	EA (n = 1613) n (%)	TA-CVS (n = 1625) n (%)	Total (n = 3238) n (%)
Bleeding	16 (1.0)	32 (2.0)	48 (1.5)
Infection	0 (0)	0 (0)	0 (0)
Spontaneous abortion	4 (0.25)	3 (0.18)	7 (0.22)
Abnormal cytogenetic result	60 (3.7)	78 (4.8)	138 (4.3)
Selective termination	57 (3.5)	74 (4.6)	131 (4.0)
Total fetal loss	61 (3.8)	77 (4.7)	138 (4.3)

**Table 3:** Randomized studies of early amniocentesis (EA) against chorionic villus sampling (CVS) or mid-trimester amniocentesis (MA), compared with the present series. GA, gestational age. Congenital anomaly and comparator values are those reported by the cited studies; the Podobnik congenital anomaly figures represent the overall series rate (1.2%).

	Nicolaides et al. 1996 [10]		Podobnik et al. 2026 [this study]		Johnson et al. 1996 [32]		Eiben et al. 1994 [16]	
	EA	CVS	EA	CVS	EA	MA	EA	MA
Number	256	262	1613	1625	344	339	2866	1802
GA range (weeks)	10 + 0–13 + 0	10 + 0–13 + 0	11 + 0–14 + 0	11 + 0–14 + 0	11 + 0–12 + 6	15 + 0–16 + 6	11 + 0–14 + 0	15 + 0–20 + 0
Chromosomal anomalies	1.8%	2.2%	3.7%	4.8%	2.3%	0.9%	2.3%	0.8%
Total fetal loss	7.9%	5.4%	3.8%	4.7%	7.8%	7.4%	4.8%	5.5%
Spontaneous loss	5.9%	1.8%	0.25%	0.18%	2.4%	3.3%	1.0%	0.5%
Congenital anomalies	4.3%	3.1%	1.2%	1.2%	2.3%	2.3%	2.2%	0.6%
Repeat testing	1.8%	2.5%	0.1%	0.2%	0.6%	0	0.2%	0
Culture failure	1.8%	0.7%	0.2%	0.3%	0.6%	0	0.3%	0.2%

**Table 4:** Maternal serum AFP and FHR recorded before and after the early invasive procedures in the 24 pregnancies with complete paired pre- and post-procedural measurements (from the 600 monitored women; 3 EA and 21 TA-CVS). Paired AFP values were available in 23 of the 24 pregnancies and paired FHR values in all 24. AFP, alpha-fetoprotein; FHR, fetal heart rate; SD, standard deviation. *p*-values are derived from paired-sample *t*-tests of the before and after values. The post-procedural rise in maternal serum AFP was statistically significant (*p* < 0.001), whereas the shift in FHR was not (*p* = 0.767); both were transient and carried no increase in the risk of miscarriage. The most pronounced AFP and FHR changes occurred in the aneuploid fetuses (trisomy 21, trisomy 13, 45,X0).

No.	AFP (ng/mL) Before	AFP (ng/mL) After	FHR (bpm) Before	FHR (bpm) After	Karyotype
1	55	147	185	220	Trisomy 21
2	75	100	125	137	Normal (46,XN)
3	35	86	144	140	Normal
4	45	92	120	115	Normal
5	77	142	145	137	Normal
6	75	125	185	213	Trisomy 21
7	51	61	125	140	Normal
8	38	48	152	140	Normal
9	65	130	129	137	Normal
10	95	125	144	135	Normal
11	77	133	147	143	Normal
12	—	—	103	97	45,X0
13	85	125	129	137	Normal
14	45	55	141	139	Normal
15	100	195	98	95	45,X0
16	23	25	137	130	Normal
17	25	42	147	145	Normal
18	43	46	135	130	Normal
19	85	115	129	130	Normal
20	65	77	144	133	Normal
21	90	110	123	113	Normal
22	85	95	155	142	Normal
23	34	42	133	130	Normal
24	65	169	207	185	Trisomy 13
Mean (SD)	62.3 (23.2)	99.3 (44.9)	140.9 (24.5)	140.1 (29.1)	
*p*		<0.001		0.767	

**Table 5:** Pulsatility index (PI) in the main uterine artery, the spiral arteries, the intraplacental arteries, the umbilical artery, the ductus venosus and the middle cerebral artery, recorded before and after early amniocentesis (EA) and transabdominal chorionic villus sampling (TA-CVS). SD, standard deviation.

Vessel (n = 400)	Before EA Mean (SD)	After EA Mean (SD)	Before CVS Mean (SD)	After CVS Mean (SD)
Uterine artery	2.10 (0.55)	2.11 (0.55)	2.11 (0.68)	2.15 (0.60)
Spiral arteries	0.60 (0.41)	0.62 (0.44)	0.65 (0.56)	0.68 (0.69)
Intraplacental arteries	0.48 (0.15)	0.51 (0.22)	0.58 (0.26)	0.60 (0.17)
Umbilical artery	2.86 (1.42)	2.88 (1.55)	2.86 (1.40)	2.90 (1.50)
Middle cerebral artery	1.88 (0.55)	1.85 (0.58)	1.88 (0.55)	1.75 (0.60)

## Data Availability

The data presented in this study are available on request from the first or corresponding author. The data are not publicly available owing to privacy and ethical restrictions.

## Abbreviations

The following abbreviations are used in this manuscript:

AFP	alpha-fetoprotein
BMI	body mass index
CEMAT	Canadian Early and Mid-trimester Amniocentesis Trial
CI	confidence interval
CNV	copy-number variant
CVS	chorionic villus sampling
DNA	deoxyribonucleic acid
EA	early amniocentesis
FHR	fetal heart rate
GA	gestational age
MA	mid-trimester amniocentesis
NIPT	non-invasive prenatal testing
PI	pulsatility index
QF-PCR	quantitative fluorescence polymerase chain reaction
SD	standard deviation
TA-CVS	transabdominal chorionic villus sampling
TC-CVS	transcervical chorionic villus sampling
